# The Clinical Course of Cirrhosis Patients Hospitalized for Acute Hepatic Deterioration: A Prospective Bicentric Study

**DOI:** 10.1097/MD.0000000000002031

**Published:** 2015-10-30

**Authors:** Yu Shi, Huadong Yan, Zhibo Zhou, Hong Fang, Jiawei Li, Honghua Ye, Wenjie Sun, Wenhong Zhou, Jingfen Ye, Qiao Yang, Ying Yang, Yaoren Hu, Zhi Chen, Jifang Sheng

**Affiliations:** From the State Key Laboratory for Diagnosis and Treatment of Infectious, Collaborative Innovation Center for Diagnosis and Treatment of Infectious Disease, The First Affiliated Hospital, Zhejiang University School of Medicine, Hangzhou, China (YS, ZZ, HF, YY, ZC, JS); Department of Infectious Diseases, Ningbo Multiple Organ Injury Research Center, Ningbo No. 2 Hospital, School of Medicine, Ningbo University, Ningbo, China (HY, WZ, JY, YH); Department of Cardiology, Ningbo Multiple Organ Injury Research Center, Ningbo No. 2 Hospital, School of Medicine, Ningbo University, Ningbo, China (HY); Department of Epidemiology and Health Statistics, Zhejiang University School of Public Health, Hangzhou, China (WS); Department of Infectious Diseases, Sir Run Run Shaw Hospital, School of Medicine, Zhejiang University, Hangzhou, China (QY); and Center for Hepatology, University College London, London, United Kingdom (JL).

## Abstract

Patients with cirrhosis are vulnerable to acute hepatic insults and are more likely to develop rapid hepatic deterioration. The aim of this study is to describe the clinical course of patients with cirrhosis and hospitalized for acute hepatic deterioration (AHD).

This is a prospective study involving 163 patients with cirrhosis and AHD. The occurrence of organ failures, systemic inflammatory response syndrome (SIRS), and infections during hospital stay were recorded and the relationship between organ failure and death or SIRS/infection was subsequently analyzed.

Of 163 patients, 35 did not develop any organ failure during in-hospital follow-ups (90-day mortality: 0%); 84 had intrahepatic organ failures (IH-OFs, defined by liver and/or coagulation failure) (90-day mortality: 22.0%); and 44 patients developed extra-hepatic organ failures (EH-OFs, defined by kidney, cerebral, circulation, and respiratory failure) on the basis of IH-OF with a 90-day mortality of 90.9%. On multivariable analysis by a Cox proportion hazard model, age, WBC, presence of IH-OF, and EH-OF all predicted 90-day death. A logistic regression analysis identified SIRS being associated with the development of EH-OF. Furthermore, IH-OF at admission and infections occurred during the hospital stay were shown to be another 2 potential risk factors.

The clinical course of cirrhosis patients with acute hepatic injury was characterized by 3 consecutive stages (AHD, IH-OF, and EH-OF), which provided a clear risk stratification. The PIRO criteria provided an accurate frame for prognostication of those patients. The systemic inflammatory response syndrome may be a target for blocking the progression to the EH-OF stage.

## Abstract 2

Supplemental Digital Content is available in the text

## INTRODUCTION

An acute hepatic insult, such as spontaneous flare-up of chronic hepatitis B (CHB), hepatitis B virus (HBV) reactivation, hepatitis A virus (HAV)/hepatitis E virus (HEV) infection, and excessive alcohol assumption, can usually lead to acute hepatic deterioration (AHD) in patients with cirrhosis.^1–7^ These patients are often at high risk of developing organ failures (OF) or even acute-on-chronic liver failure (ACLF), which is associated with high short-term mortality.^8,9^ Our recent study has demonstrated that although intrahepatic organ failures (IH-OF, defined as liver and/or coagulation failure) were more frequently seen in those patients, some patients also developed extra-hepatic organ failures (EH-OF, defined as cerebral, kidney, respiratory, and circulation failure).^10^ Therefore, abnormality in liver and coagulation system could be regarded as the early and direct consequence resulting from an acute hepatic insult, whereas the occurrence of extra-hepatic organ failures may be a delayed event, which indicates a more severe stage of disease.

However, the question regarding to the causal factors for extra-hepatic organ failures in this population of patients remains unresolved. One possible precipitating factor is systemic inflammatory response (SIRS), which consists of heart and respiratory rate, white cell count and temperature. It has been shown that SIRS is associated with hepatic encephalopathy, renal failure, and poor outcome in patients with acute liver failure or cirrhosis.^5,11,12^ In addition, the cirrhosis patients were at high risk of being complicated with bacterial infection^5,10^ and there is strong evidence that bacterial infection can lead to organ failures and significantly increased mortality in patients with liver cirrhosis.^13^

To describe the clinical course of this population of patients, a prospective cohort of 163 patients with liver cirrhosis and acute hepatic deterioration (AHD) were recruited from 2 clinical centers, and the occurrence of organ failures was recorded during the overall in-hospital period. Additionally, the association between SIRS/infection and development of extra-hepatic organ failure was also explored in this study.

## STUDY POPULATION AND METHODS

### Study Population

Patients with acute hepatic injury were identified in a registered prospective cohort of patients with liver cirrhosis (ChiCTR-OCH-14005018), who enrolled from May 1, 2014, and February 25, 2015, in hepatic failure ward of First Affiliated Hospital of Zhejiang University and Ningbo No. 2 Hospital. The diagnosis of cirrhosis was described by previous studies:^10,14^ by liver biopsy, endoscopy, radiological examination, or clinical evidence of prior hepatic de-compensation. Acute hepatic injury (AHI) was defined as a rise of alanine aminotransferase (ALT) ≧5 times of the normal upper limit or more than twice of the baseline value within 1 month. Acute hepatic deterioration (AHD) was defined on the basis of AHI and should meet the following criteria as previously described: increase of serum bilirubin ≧85 μmol/L and international normalized ratio (INR) ≧1.5 with a definite hepatic insult.^10^ As previously described, hepatic-related acute precipitating events included spontaneous flare-up of CHB, HBV reactivation, HAV and HEV superimposed infection, hepatotoxic drug use, and active alcohol drinking. Among them, spontaneous flare-up of CHB was defined as a flare-up of ALT ≧5 times of the normal upper limit, and/or more than twice of the baseline value, with replicating HBV-DNA (10E^5^ copies/mL for HBeAg-positive patients; 10E^4^copies/mL for HBeAg-negative patients) within 1 month before admission. HBV reactivation was defined as reappearance of serum HBV-DNA along with a flare-up of ALT ≧5 times of the normal upper limit, and/or more than twice of the baseline value in a person known to have the inactive HBsAg carrier state or resolved hepatitis B. Active alcohol drinking-related AHI should have aspartate aminotransferase (AST) to alanine transaminase (ALT) ratio > 1 (AST/ALT>1), in addition to a flare-up of ALT and excessive alcohol assumption within 1 month.

Patients were excluded based on the following criteria: (i) age<18 years; (ii) pregnancy; (iii) hepatocellular carcinoma (HCC) outside Milan criteria or other types of tumors; (iv) previously received a liver transplant; (v) HIV infection.

This study complies with the principles of the Declaration of Helsinki and was approved by the ethics committee of both First Affiliated Hospital of Zhejiang University and Ningbo No. 2 Hospital. Written consents were obtained from each participant or their legal representatives.

### In-Hospital and Out-Patient Data

At admission, patients were evaluated for the presence of organ failure and SIRS. For patients suspected for infection, an initial screening by laboratory blood and urine tests, pulmonary CT scan, and abdominal ultrasound was performed, followed by cultures of blood, urine, sputum, or ascites (as well as ascitic fluid analysis). For in-hospital follow-up, the presence and development of organ failure and SIRS were checked at least every 3 days. In-hospital infection was also screened for suspected patients. In-hospital death was recorded. For those discharged from hospital, 28-day and 90-day prognosis were recorded, which were verified by medical records, telephone contact, or visiting.

Organ failure was categorized into either intrahepatic or extra-hepatic identities and the definition was based on the chronic liver failure-sequential organ failure assessment (CLIF-SOFA) (9) (see Table 1). Systemic inflammatory response is defined as at least 2 of the following components: core temperature of >38 °C or <36 °C; heart rate >90 bpm; respiratory rate >20 bpm; white blood cell (WBC) count >12,000/mm^3^.^15^ The presence of SIRS was confirmed by at least 2 repetitive measurements with at least 2 h interval. Bacterial or fungal infections were diagnosed as previously described:^14^ (1) pneumonia: new pulmonary infiltrate with fever, respiratory symptoms, findings on auscultation, or WBC count >10,000/mm^3^ or <4000/mm^3^; (2) spontaneous bacterial peritonitis (SBP): polymorphonuclear cells in ascitic fluid >250/μL; (3) bacteremia: positive blood cultures without a source of infection; (4) urinary tract infection: urine WBC >10/high power field with positive culture and symptom of urinary irritation; (5) others bacterial infections included skin infection and intra-abdominal infection; (6) fungal infection: positive fungal culture.

**TABLE 1:**
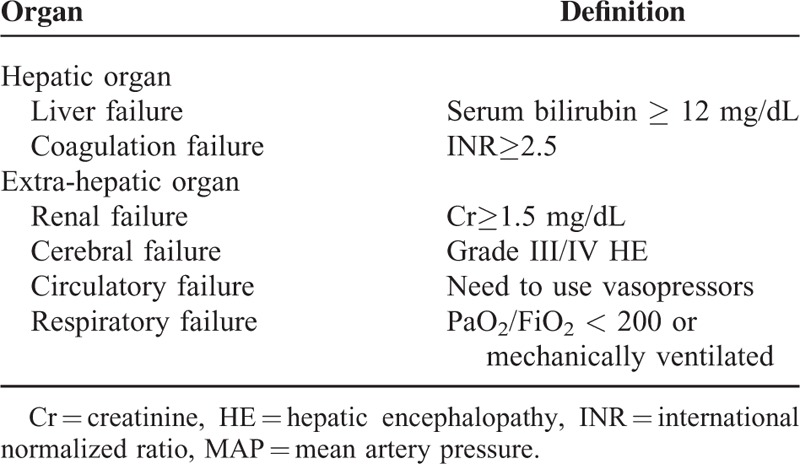
Definition of Organ Failure

Patients with detectable serum HBV-DNA were treated with nucleoside analogs (lamivudine alone 100 mg, telbivudine alone 600 mg, entecavir alone 0.5 mg, or lamivudine 100 mg plus adefovir 10 mg daily). Patients with bacterial or fungal infection were treated with intravenous albumin plus antibiotic or antifungal therapy empirically or based on the culture test. Patients with overt ascites were treated with diuretics (moderate) or paracentesis plus intravenous albumin (large or refractory). Patients with renal failure were treated with intravenous albumin with or without vasoconstrictors (such as dopamine and terlipressine). Patients with hepatic encephalopathy were treated with lactulose, antibiotics, and l-ornithine aspartate. Patients with circulation failure were treated with fluid replacement, followed by vasoactive agents. Patients with respiratory failure were treated with oxygen therapy (mask or venturi mask oxygen inhalation) or mechanical ventilation. Patients with coagulation failure received plasma transfusion. Patients with liver failure were treated with ademetionine, ursodesoxycholic acid, glycyrrhizic acid, or artificial liver support. Patients with hypo-albuminemia were given intravenous albumin.

### Statistical Analysis

Variables were expressed as mean ± standard deviation (SD), median with interquartile range, or number/percentage. Continuous variables were compared using Mann–Whitney *U* test and nominal variables were compared via chi-square test. The log-rank test was used to compare survival among/between the groups and survival curves were drawn by GraphPad Prism 5 (GraphPad Software, San Diego, CA). To identify independent risk factors associated with time-dependent death of patients, a bi-variate analysis using the Cox proportional hazard model was performed to screen candidate variables (*P* < 0.10), followed by multivariate analysis. A multivariate logistic regression analysis was performed to explore predictors for development of extra-hepatic organ failures. For multivariate analysis, the entry and removal probability for stepwise was set as 0.05 and 0.10, respectively, and variables with *P* < 0.05 were kept in the final model. All the statistical analyses were performed with SPSS version 16.0 (SPSS Inc, Chicago, IL).

## RESULTS

**FIGURE 1:**
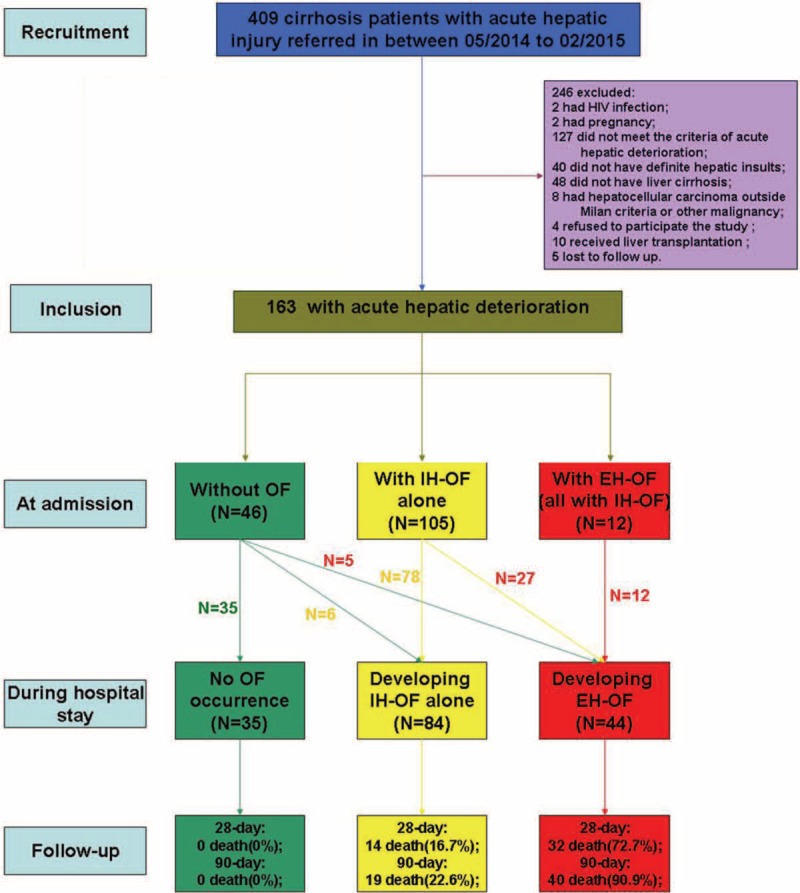
Flowchart of patient selection and follow-up. AHI = acute hepatic injury, EH-OF = extra-hepatic organ failure, HCC = hepatocellular carcinoma, IH-OF = intrahepatic organ failure, OF = organ failure.

As shown in Figure 1 and Table 2, a total of 163 patients with cirrhosis and acute hepatic deterioration were studied prospectively. Out of the total population, 79.8% was men, with a mean age of 50.5 ± 12.2 years, and most patients had (93.9%) underlying HBV-related cirrhosis. Among various precipitating events, spontaneous flare-up of CHB (68.7%) and HBV reactivation (19.0%) were the commonest, and ascites (74.8%) was the commonest complication, followed by hepatic encephalopathy (12.9%). A total of 19.6% of patients had bacterial or fungal infection, and 15.3% of patients had SIRS. The mortality at 28-day and 90-day was 28.2% and 36.2%, respectively.

**TABLE 2:**
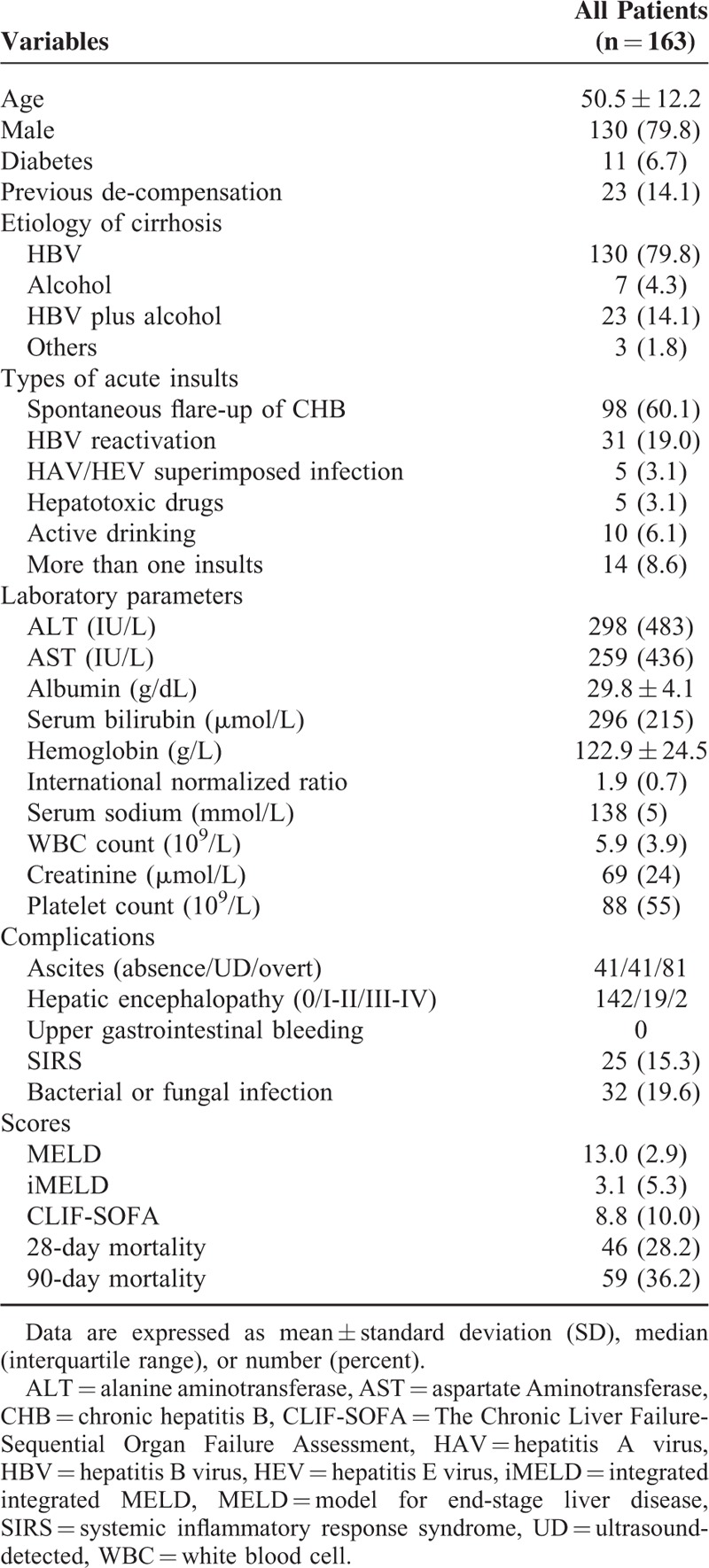
Demographic and Clinical Characteristics of Patients in the Cohort

### Evolving Organ Failures During Hospital Stay

It was observed that 117 of 163 patients had organ failures at admission. Among them, most patients (89.7%) had intrahepatic organ failure (liver and/or coagulation failure) alone (see Fig. 1). During the hospital stay, another 32 patients developed extra-hepatic organ failures, of which 84.4% already had IH-OF at admission. Of interest is that the 5 patients who did not have IH-OF at admission, progressed to EH-OF, and also developed IH-OF after admission (data not shown). The specific types of organ failures occurred at admission or during the entire in-hospital period were listed in Table 3.

**TABLE 3:**
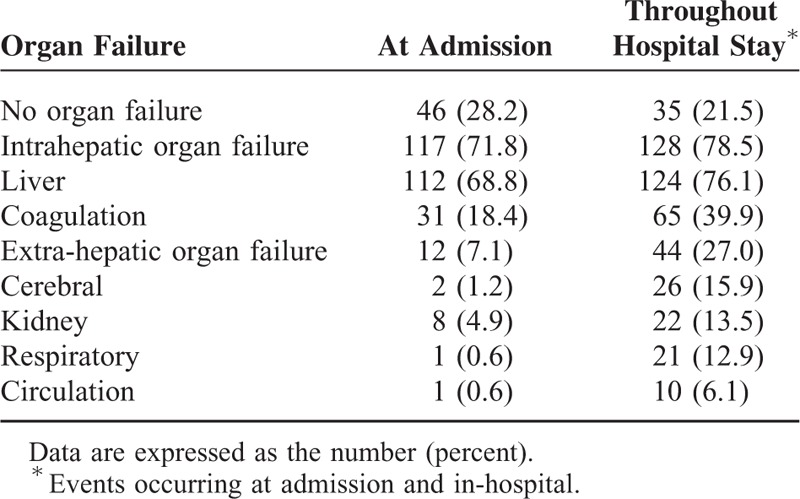
Episodes of Organ Failures in 163 Patients at Admission and Throughout Hospital Stay

### The Relationship Between Organ Failures and Transplant-Free Mortality

**FIGURE 2:**
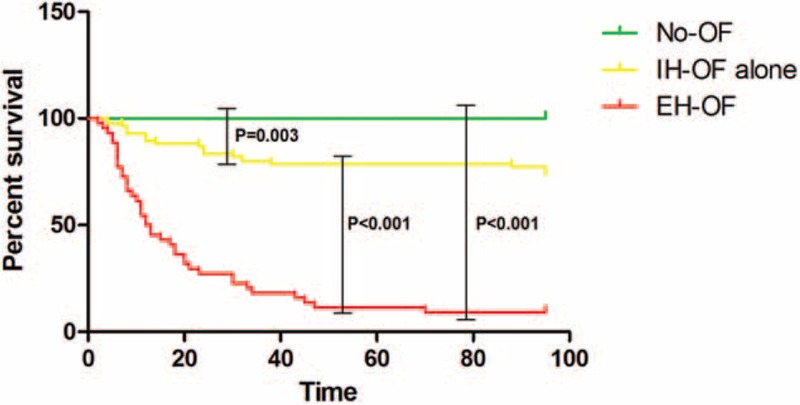
Comparison of 90-day survival among No-OF, IH-OF, and EH-OF groups. The cumulative survival across the groups was compared using the log-rank test. EH-OF = extra-hepatic organ failure, IH-OF = intrahepatic organ failure, OF = organ failure.

Patients, who developed EH-OF, had extremely high 28-day (72.7%) and 90-day mortality (90.9%). Similarly, patients who solely had IH-OF, within in-hospital follow-up, also had a considerable 28-day and 90-day mortality at 16.7% and 22.6%, respectively. In contrast, those with no organ failure had a more favorable outcome, with no death reported within 90-day follow-up (see Fig. 1). The significant difference on 90-day cumulative survival among the 3 groups is shown in Figure 2.

To further confirm the observation, a Cox proportional hazard model was used to analyze the association between organ failure and short-term mortality (a preliminary screening analysis was seen in supplementary Table 1, http://links.lww.com/MD/A520). According to the admission data, elder age, high WBC count, presence of IH-OF and EH-OF were all independently associated with 90-day mortality on multivariable analysis; however, after combining with the in-hospital data, only elder age, presence of IH-OF and EH-OF were shown to be significant predictors (see Table 4). Of particular interest is that EH-OF was the strongest variable in predicting short-term mortality.

**TABLE 4:**
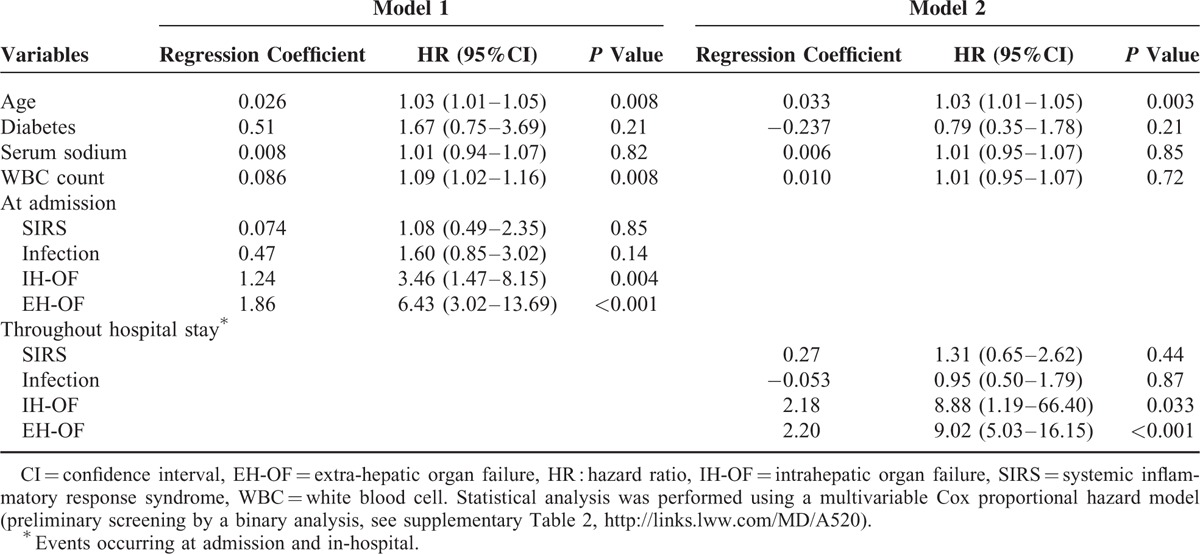
Multivariate Analysis of Independent Risk Factors of 90-Day Mortality by Cox Proportional Hazard Model

### The Relationship Between SIRS/Infection and Extra-Hepatic Organ Failures

**FIGURE 3:**
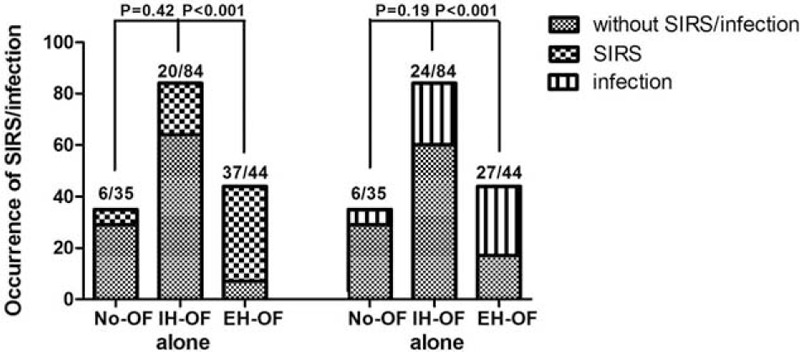
Occurrence of SIRS and infections in No-OF, IH-OF, and EH-OF groups throughout hospital stay. The statistical analysis was performed using the chi-square test. EH-OF = extra-hepatic organ failure, IH-OF = intrahepatic organ failure, OF = organ failure, SIRS = systemic inflammatory response syndrome.

We next explored the factors related to the development of EH-OF. We found patients with EH-OF had significantly higher occurrence of SIRS and infection compared to those with IH-OF alone, or those without OF at admission or during the in-hospital period (see Fig. 3). Furthermore, a multivariable logistic regression analysis showed that the presence of SIRS and IH-OF at admission predicted the development of EH-OF (a preliminary screening analysis was seen in supplementary Table 2, http://links.lww.com/MD/A520). By using in-hospital data, in addition to SIRS, infection was a potentially significant predictor (see Table 5).

**TABLE 5:**
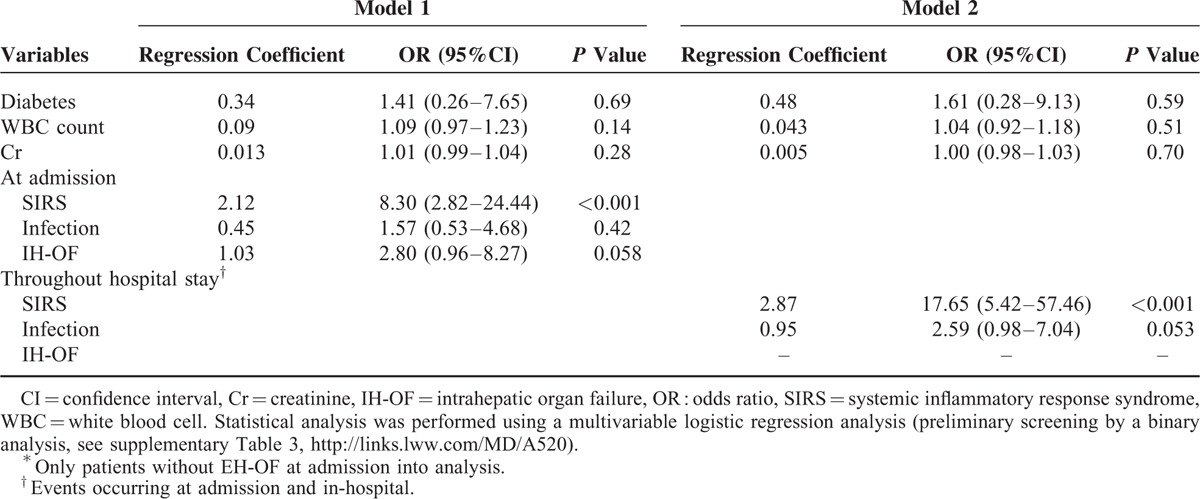
Risk Factors Associated With Development of Extra-hepatic Organ Failures by Multivariate Logistic Regression Analysis^∗^

**FIGURE 4:**
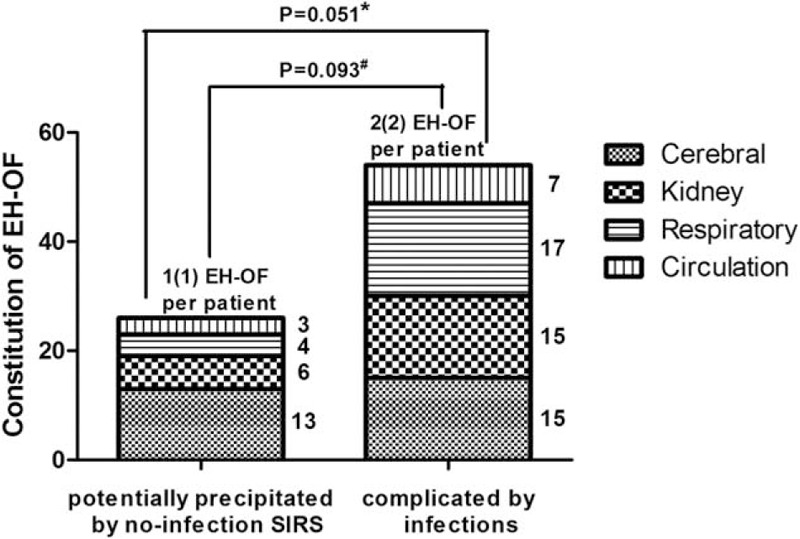
Comparison of number and type of EH-OF among those developing EH-OF according to potential precipitating factors. Asterisk (^∗^) indicates the comparison of the constitution of EH-OF (cerebral and noncerebral) between the 2 groups. Hash (^#^) indicates the comparison of EH-OF number per patient between the 2 groups. EH-OF = extra-hepatic organ failure.

Among patients who developed EH-OF, we further compared the types and number of EH-OF between those complicated with infection and those without. We found the constitution of EH-OF in patients precipitated by no-infection SIRS seemed to be dominated by cerebral failure, whereas patients with infection had a more complicated representation of EH-OF, although it did not reach statistical significance. It was also observed that patients complicated with infection had higher numbers of EH-OF per person than those without (see Fig. 4).

The detailed information of infection was listed in Table 6. Nearly all infection was acquired in-hospital or health-care related. Out of which, the commonest type of infection was pneumonia, followed by SBP and bacteremia. Totally 23.2% of infection was culture-confirmed, with a considerably high occurrence of fungal infection. The information of isolated pathogen was listed in Table 7.

**TABLE 6:**
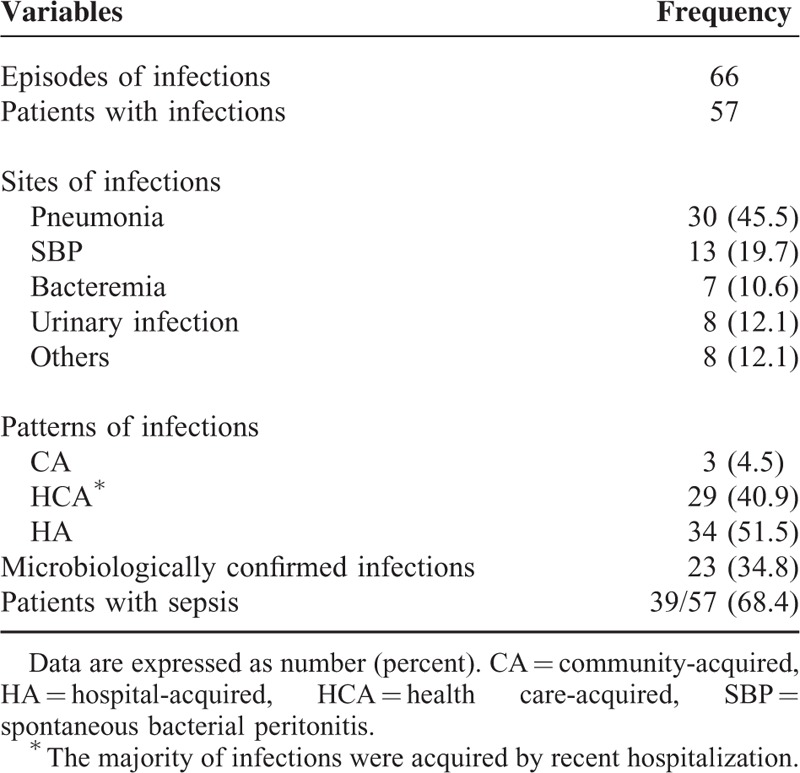
Detailed Information of Infections Reported During Overall In-Hospital Period

**TABLE 7:**
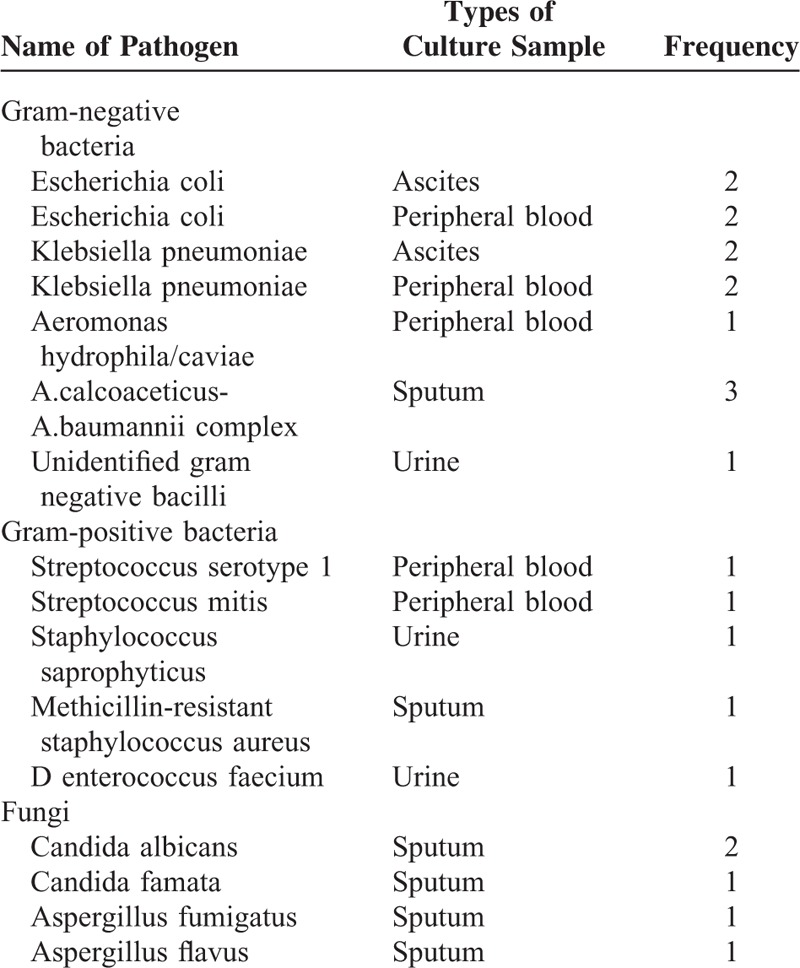
List of Isolated Pathogen

### A Proposed Model for the Natural History of Cirrhosis Patients With Acute Hepatic Deterioration

**FIGURE 5:**
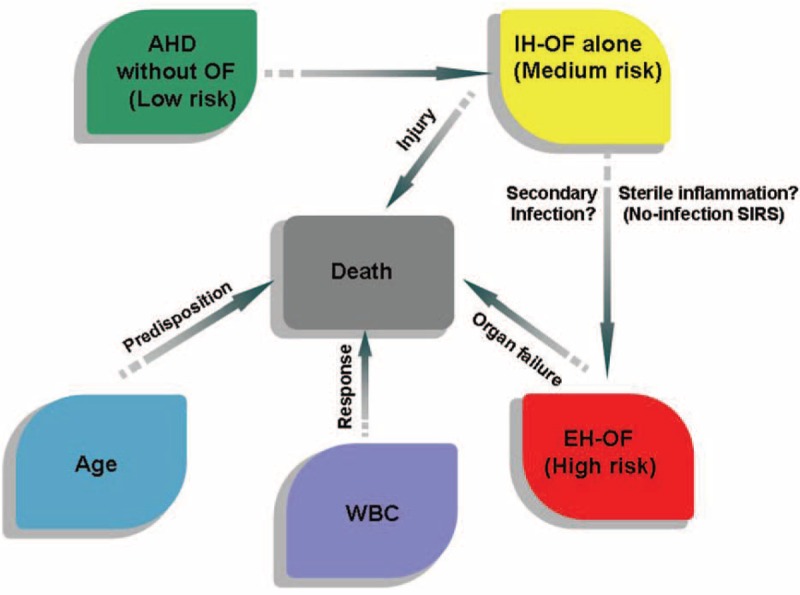
The graphic scheme of the natural history of cirrhosis patients with acute hepatic injury. AHD = acute hepatic deterioration, EH-OF = extra-hepatic organ failure, IH-OF = intrahepatic organ failure, OF = organ failure, SIRS = systemic inflammatory response syndrome, WBC = white blood cell.

Based on the above findings, we propose a model for delineating the natural history of this special population (see Fig. 5). A consecutive 3-stage categorization, including acute hepatic deterioration (AHD), IH-OF and EH-OF, was used for dividing the clinical course. Patients remaining at the AHD stage were likely to be safe, but progression to IH-OF could lead to higher risk of EH-OF development and medium risk of death in patient. Therefore, extra cautions should be taken at this stage. Inflammation and/or infection might be potential precipitating factors in the development of EH-OF, and the factors determining 90-day death fit well with PIRO criteria: age (predisposing), IH-OF (injury), WBC (response), and EH-OF (organ failure).

## DISCUSSION

Building upon the previous studies,^2,5,16,17^ this study further described the clinical characteristics of cirrhosis patients precipitated by severe hepatic injury. Precipitated by acute hepatic insults, the patients represented an acute exacerbation of hepatocellular function, manifesting as severe jaundice and coagulaopathy at early stage of disease onset. Additionally, the patients usually had relatively well compensated cirrhosis before the attack. Moreover, the patients with poor prognosis usually developed extra-hepatic organ failures at late stage. Lastly, most death occurred within 90 days of admission or diagnosis, and patients who recovered from this attack had relatively favorable long-term outcome^10^ (Abdul-Hai et al, 2015).

Our study highlighted several important findings: we observed that organ failures occurred in a chronological order: almost all patients had liver and/or coagulation failure before developing extra-hepatic organ failures. Although abnormality in the coagulation system could be caused by sepsis, it would be reasonable to regard it as another reflection of liver failure in this clinical setting, as it occurred simultaneously with jaundice and independently of infections. Further analysis showed that the presence of liver/coagulation failure was a strong predictor of extra-hepatic organ failures. Moreover, patients with EH-OF had a significantly higher short-term mortality than those with IH-OF alone. Thus, these findings suggest that IH-OF and EH-OF were 2 consecutive stages of disease progression, and such phenomenon was characteristic of cirrhosis patients with acute hepatic deterioration (AHD), which could not be observed in infection-precipitated ACLF patients.^18^

This study also reported that infection in AHD patients with cirrhosis was different from that of the overall population of patients with liver cirrhosis. Infections were seldom acquired from community, suggesting that the acquirement of infections was a late event in the natural history of patients with acute hepatic injury. Secondly, pneumonia but not SBP was the commonest type of infection, perhaps due to most patients having well-compensated cirrhosis previously. The occurrence of fungal infection was not rare, which might be because of long-term antibiotic treatment or immune paracensis.^19,20^

In a recent study, our study showed a relationship between SIRS/infection and EH-OF.^5^ The finding was not surprising because the importance of SIRS has been highlighted in patients with cirrhosis, which is associated with renal dysfunction, worsening of hepatic encephalopathy, and poor in-hospital outcome.^21,22^ Infection has also been demonstrated to lead to multiorgan failures.^23,24^ It should be noted that although infections accounted for a large proportion of SIRS, some cases of SIRS occurred independently from SIRS. Both SIRS with infection (sepsis) and without (sterile inflammation) were associated with EH-OF development (data not shown), suggesting that both sterile and infection-related systemic inflammation might be important precipitants of disease progression. According to our previous study, ACLF patients precipitated by infections had higher occurrence of EH-OF than those precipitated by hepatic insults.^10^ In line with it, our results indicated a trend toward higher numbers of EH-OF per patient in patients with infections. The findings suggest that secondary infections complicated the natural history of AHD patients with cirrhosis. Of course, the causal relationship between SIRS/infection awaits more definitive confirmation and further studies are required to clarify this issue.

The PIRO criteria have been successfully applied to predict the prognosis of ACLF patients.^25^ In line with the criteria, 4 predictors of poor prognosis were found to be age, IH-OF, WBC, and EH-OF, which fit well with the corresponding PIRO components (predisposition, injury, response, and organ failures). The value of age in predicting the prognosis of cirrhosis patients with hepatic injury has been reported in our previous studies^10,26^ and might be linked to the decreased tolerance to damage. WBC, reflecting body inflammatory response to insults, has been confirmed to be a strong predictor of poor prognosis in cirrhosis patients with acute de-compensation.^9,27^

Liver transplantation (LT) is the major therapeutic option for patients with acute hepatic deterioration; however, the decision should be made by weighing the need for LT and the risks associated with the procedure.^28^ For patients with IH-OF, LT should be considered for those with concurrent SIRS as these patients are more susceptible to developing EH-OF. Previously, 2 studies showed an encouraging long-term prognosis (a 5-year survival of 70–80%) in ACLF patients,^29,30^ suggesting that liver and coagulation failure had little impact on post-operation mortality. However, some of the patients with uncontrolled infection-induced SIRS would be less likely to receive LT. For patients with EH-OF, the possibility of LT should be considered for all the patients because of the extremely high short-term mortality. Previous studies showed cerebral (hepatic encephalopathy), kidney, and pulmonary failure were not absolute contraindication for LT, although likely to decrease the success of LT.^30–32^

The present study still has certain limitations. Some cases did not progress to EH-OF when discharged from the hospital due to nonmedical reasons. In these patients, the role of EH-OF might be underestimated in the study and a prolonged follow-up is needed. Also, the diagnosis of infections might be underestimated using the current criteria and some cases of SIRS might be attributed to occult infections. In addition, the causal relationship between SIRS/infections could not be established in the study and the majority of our study population had HBV-related cirrhosis and precipitated by spontaneous flare-up of CHB and HBV reactivation.

Based on the above findings, the present study proposes a categorization of the natural history of patients with cirrhosis and acute hepatic deterioration by 3 consecutive stages of AHD, IH-OF, and EH-OF. Such categorization effectively described the dynamic disease progression of those patients and provided clear risk stratification, therefore, provides clinical implications for designing therapeutic strategies. For patients in the IH-OF stage, infection control (or prevention) and resolving inflammation might be 2 separate therapeutic targets, and it is crucial to prevent the development of EH-OF. However, once patients developed EH-OF, the possibility of liver transplantation should be immediately considered.
